# Development of human prostate cancer stem cells involves epigenomic alteration and PI3K/AKT pathway activation

**DOI:** 10.1186/s40164-020-00168-0

**Published:** 2020-06-11

**Authors:** Jingjing Wu, Shundong Cang, Christina Liu, Whitman Ochiai, Jen Wei Chiao

**Affiliations:** 1 Department of Oncology, The First Affiliated Hospital of Zhengzhou University, Zhengzhou, Henan Province China; 2 Department of Oncology, The Henan Province Hospital of Zhengzhou University, Zhengzhou, Henan Province China; 3 Northwestern University, Evanston, IL 60201 USA; 4 Department of Medicine, New York Medical College, Valhalla, NY 10595 USA

**Keywords:** Cancer stem cells, Prostate cancer, Sphere, PI3K, AKT, Histone acetylation

## Abstract

**Background:**

Human prostate cancer spheres endowed with stem cell properties have been obtained from androgen-dependent cell line LNCaP after exposure to an epigenomic modulator phenethyl isothiocynate (PEITC). Sphere cells can self-renew and grow with androgen, and also without androgen. Little is known about the signaling pathway and mechanism in the development of the stem cells in the spheres.

**Methods:**

Expression of phosphoinositol-3 kinase (PI3K) pathway members and histone acetylation were quantified in the tumor spheres and LNCaP cells by western immunoblotting.

**Results:**

The level of phosphorylated AKT was significantly increased in the sphere stem cells than the LNCaP cells at an average of 7.4 folds (range 5.8–10.7 folds), whereas the P27 level was elevated 5.4 folds (range 4.8–6.3 folds) (*P *< 0.05). The acetylation level on histone H3 lysine 9 was decreased.

**Conclusions:**

PEITC appears to regulate the epigenome through histone acetylation and activate the PI3K/AKT pathway in the LNCaP cells. This mechanism may be responsible in part for the development of the prostate cancer stem cells.

## Background

Prostate cancer remains a worldwide challenge, particularly in the developed countries with increased screening [1–3]. Androgen-dependent prostate cancer recurs when the residual cells become androgen-independent and hormone refractory [4–6]. In an earlier study we identified prostate cancer stem cells (PSC) that form spheres in a cell culture system mimicking the evolving process of prostate tumors from an androgen-dependent to androgen-independent state [7]. The spheres were isolated from the androgen-dependent LNCaP cell culture after exposure of the cells to phenethyl isothiocyanate (PEITC), an epigenomic modulator [8–11]. The sphere cells were shown to be endowed with PSC properties and virtually perpetuate in the culture system. The spheres can grow with androgen, and also without androgen. They can differentiate into tumor cells with neuroendocrine properties in the absence of androgen, and can reverse to spheres when androgen is supplied. The plasticity of the PSC highlights their adaptability in different conditions to grow, differentiate, and survive. We hypothesize that these PSC cells may be responsible for prostate cancer resistance to hormonal therapy [7].

The PEITC is a type of isothiocyanates that are present naturally in cruciferous vegetables [8, 12]. It is an epigenomic modulator that mediates histone modifications and gene expression [10, 11, 13, 14]. The detailed molecular mechanisms involved in the tumor stem cell development however remain elusive.

The phosphoinositol-3 kinase (PI3K) signaling pathway is one of the most well-known cancer survival pathways and plays a crucial role in cancer cell proliferation and differentiation [15–17]. The PI3K downstream signaling pathway involves activation of AKT, a protein kinase B [15, 18]. The PI3K pathway enzymes are also components of the insulin signaling pathway [19]. When PI3K pathway becomes overactive in cancer cells it increases glucose intake and fuels the cancer cell growth [20–22].

In this study, we examined the activities of the PI3K signaling pathway members such as AKT, P27, and the level of histone acetylation. The results showed that the PI3K pathway members were more frequently activated in the sphere PSC than in LNCaP cells. Increase in the level of phosphorylated AKT (p-AKT), a critical PI3K pathway member correlated to a decrease in histone acetylation.

## Materials and methods

### Cell lines and cell cycle analyses

Human androgen-dependent prostate cancer cell line LNCaP was purchased from ATCC and maintained for fewer than 4 months before experimentation. A permanent culture of the PSC spheres was established according to previously described procedure [7]. LNCaP cells and the PSC spheres were maintained in RPMI-1640 medium supplemented with 10% fetal bovine serum and 1% antibiotics.

Distribution of cell cycle phases was measured by a BD FACS Calibur flow cytometer with established procedures [8, 23]. Cells were first fixed with 80% ethanol at 4 °C, and incubated on ice followed by propidium iodide (50 µg/ml) staining of the DNA.

### Immunoblotting

The levels of cellular protein expression were determined by quantitative Western blotting as previously described [24, 25]. Images of immunoprecipitation were revealed using chemo Imager 5500 (Alpha Innotech). Western blotting was also performed with capillary electrophoresis-western blot by RayBiotech, Inc. (Norcross, GA, USA) using a WES capillary electrophoresis device (ProteinSimple, San Jose, CA, USA). Approximately 40 nL lysate from each sample was injected into the capillary electrophoresis device, and the proteins were separated by size through a stacking and separation matrix in the capillary which were immobilized to the capillary wall. Matrix was removed and individual primary antibody passed through the capillary. A HRP-conjugated second antibody (ProteinSimple 042-205) was used along with a chemiluminescent substrate (ProteinSimple PS-CS01) for image development. For each protein analysis, a sample containing the known protein was subjected identically to the capillary electrophoresis-western blot procedure as a positive control for the primary antibody, and as a reference to the protein molecular weight. The intensity of the chemiluminescence from each protein blot was quantified and presented as intensity peak. The mean values of two groups of data were compared by the two-tailed Student’s t-test, with *P *< 0.05 considered to be statistically significant.

## Results

### Cell cycle and histone acetylation in prostate cancer stem cells

**Fig. 1:**
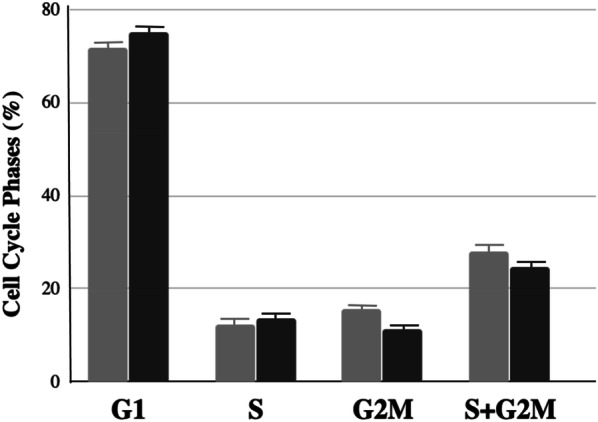
Cell cycle analysis of prostate tumor spheres and LNCaP cells. Cell cycle phase distribution in G1, S, G2M, and S + G2M was analyzed by flow cytometry study of LNCaP cells (grey color column) and sphere cells (solid bar). Columns and vertical bars are means and ranges from independent experiments

To investigate the mechanisms involved in the PSC sphere formation, the status of cellular proliferation of the sphere cells was first examined. Cell cycle phase distribution was determined with a flow cytometric method. The spheres and LNCaP cells were obtained from their respective cultures 24 h after medium change. The graph in Fig. 1 reveals the different proportions of the S and G2M cell cycle phases between the LNCaP cells and spheres. The total replicating sphere cells (S + G2M) was 24.9%, less than that of the LNCaP cells which had 28.2%. The analyses indicated that the sphere cells grew slower than the LNCaP cells.

**Fig. 2:**
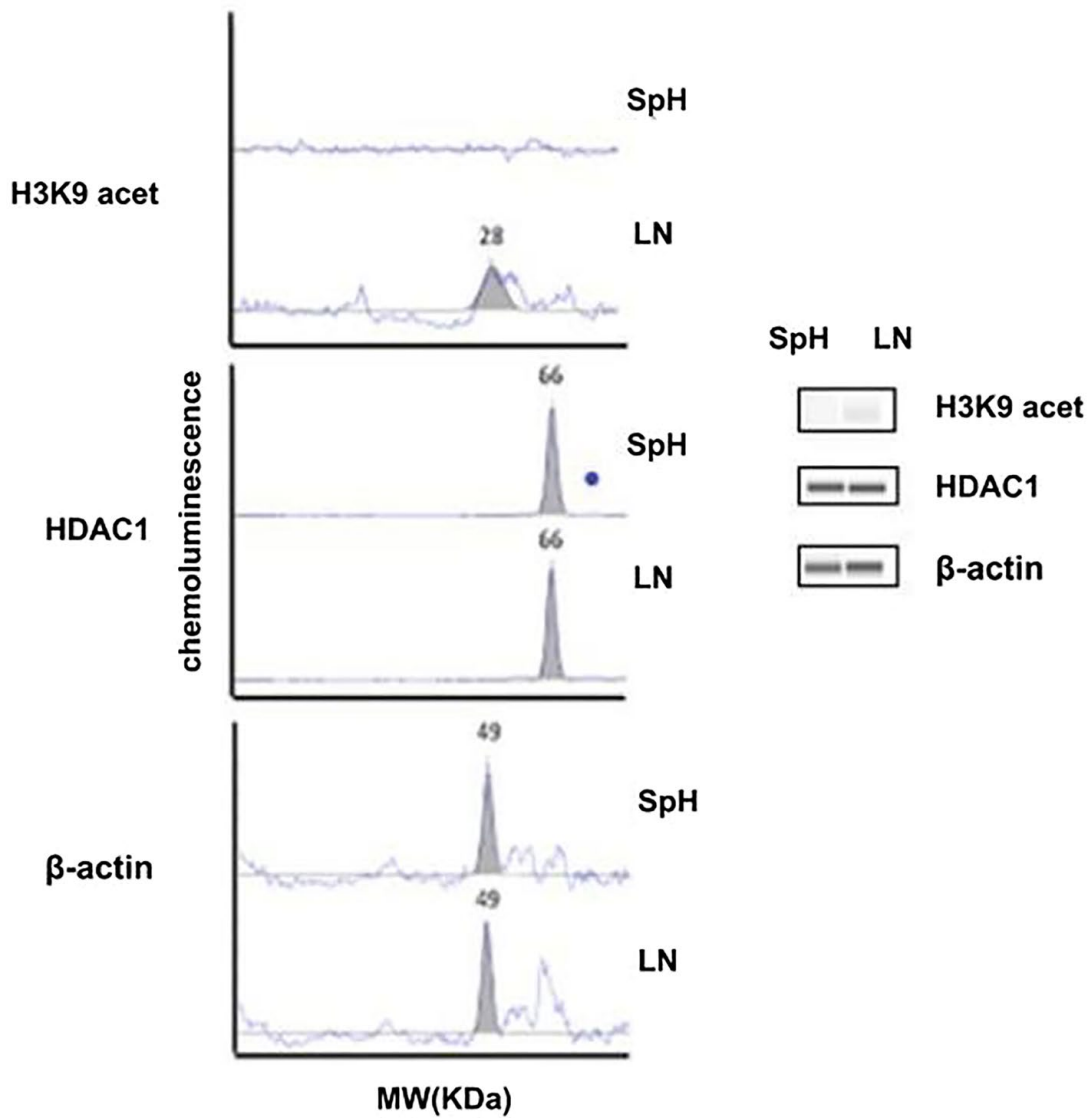
Histone H3 acetylation and HDAC1 expression in the sphere and LNCaP cells. Capillary electrophoresis-western blot analysis was used to assess the level of acetylated H3K9 and HDAC1 expression. The electropherogram of acetylated H3K9, HDAC1, and β-actin proteins from the lysates of the LNCaP cells (LN) and spheres (SpH) is shown in the left panel, where Y axis represents the chemiluminescence and the X axis molecular weight (kDa). The right panel of the figure shows the Western blot images of H3K9, HDAC1 and β-actin. The β-actin was used as a loading control

Since PEITC is a known epigenomic modulator, the levels of histone acetylation and histone deacetylase enzyme HDAC1 were evaluated. The acetylation level on histone H3 lysine 9 (H3K9) was quantified with a capillary electrophoresis-based western blot system. The immune precipitation with anti-H3K9 was quantified with chemiluminescence and expressed as peak area, as shown in Fig. 2. The graph shows that H3K9 acetylation was not detectable in the spheres, in contrast to the LNCaP cells which had demonstrable H3K9 acetylation. These results indicated a significant reduction of acetylation of H3K9 in the spheres. Figure 2 revealed further that there was no difference in the HDAC1 expression level between the spheres and LNCaP cells. The results revealed that compared to the LNCaP cells, the prostate cancer stem cells may have acquired a unique histone acetylation pattern.

### PI3K pathway in prostate cancer stem cells

**Fig. 3:**
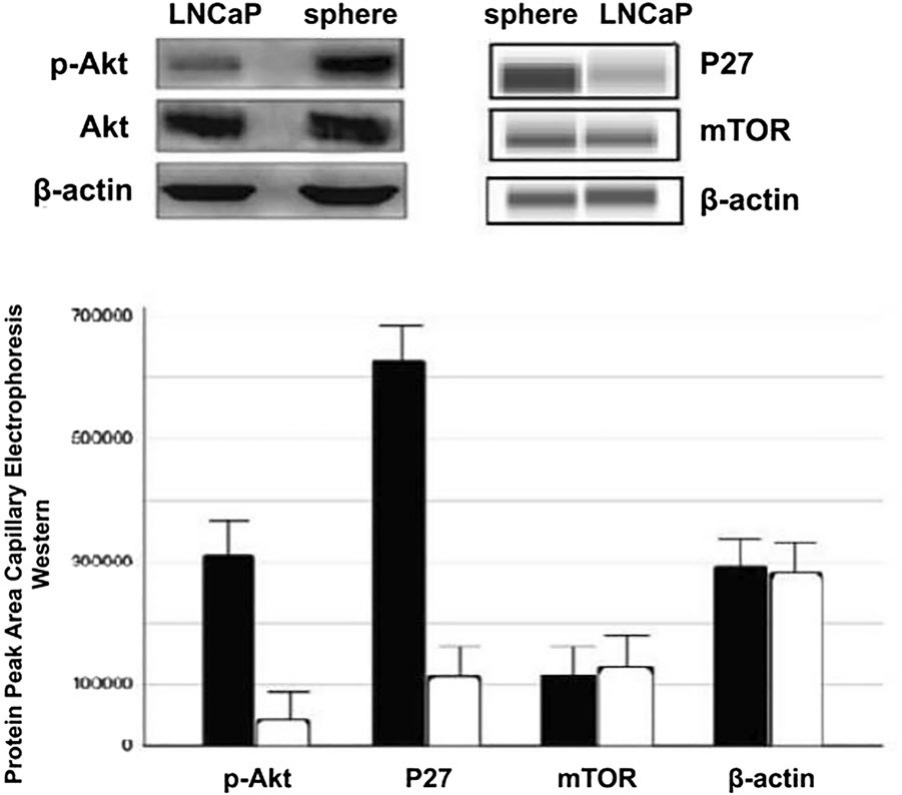
Analysis of phosphoinositol-3 kinase pathway members. Upper left panel: representative blots of p-AKT and AKT expression in the LNCaP cells and the spheres. β-actin was used as a loading control. Upper right panel: representative blots from capillary electrophoresis-western blot of protein p27 and mTOR from the spheres and the LNCaP cells. β-actin was used as a loading control. The bar graph in the lower panel depicts quantity of the protein expression in chemiluminescence units (area under peak) for each protein assayed by the capillary electrophoresis-western blot analysis. Solid bars indicate the values of the spheres, and open bars the values of LNCaP cells. Columns and vertical bars are means and ranges from independent experiments. The levels of p-AKT and P27 in the spheres and LNCaP cells were statistically significant (*P *< 0.05)

The spheres and the LNCaP cells were evaluated for the expressions of critical mediators of PI3K pathway by Western blotting. The blot images in the upper left panel of Fig. 3 showed that the level of phospho-AKT (p-AKT) in the sphere cells was clearly higher than that of the LNCaP cells. Quantitative change of p-AKT was further evaluated by capillary electrophoresis western blotting, with the chemiluminescence of immunoblots quantified as area under peak (lower graph, Fig. 3). The p-AKT level was determined to be significantly increased at an average of 7.4 folds (ranged 5.8-10.7 folds) over the level of LNCaP cells (*P *< 0.05).

The expression level of P27 and mTOR in the LNCaP cells and the spheres was also quantified with capillary electrophoresis-western blotting. Representative images were displayed in Fig. 3 upper right panel. The P27 expression was significantly increased by 5.4–folds (ranged from 4.8 to 6.3 folds) in the spheres, as compared to that of the LNCaP cells (P < 0.05). The mTOR expression was unchanged between the LNCaP cells and spheres, as the ratio of the mTOR expression in the spheres over LNCaP was 1.1 (Fig. 3, bar graph).

## Discussion

This study revealed that activation of the PI3K pathway members as well as epigenomic modifications may be the mechanisms underlying the development of the prostate cancer stem cells from LNCaP cells. The biological effects of AKT phosphorylation and activation of the downstream PI3K signaling pathway play an important role in the prostate carcinogenesis [26]. This pathway is know to be overactive in cancer cells in general by promoting growth and reducing apoptosis [27]. In clinical specimens, the phospho-AKT quantity was more abundant in high-risk prostate cancers with Gleason grades 8-10 than those less aggressive prostate cancers [28].

Activated AKT mediates downstream responses by phosphorylating a range of proteins [15, 16]. Erdogan et al. [29] evaluated the effects of a flavonoid quencetin on the proliferation of stem cell spheroids isolated from prostate tumor PC3 and LNCaP cell lines. They reported that the mechanism responsible for inhibiting the stem cell proliferation involved inhibition of AKT phosphorylation. Another study using isolated PC3 tumor stem cells demonstrated that the PI3K and p-AKT protein levels in the PC3 cells were decreased after treatment with apigenin, an agent that inhibits the tumor growth [30]. Similarly, Dubrovska et al. [31] described that phospho-AKT level was increased in the PC3 and DU145 sphere cells, as compared to that in the parental PC3 and DU145 cells. In addition, AKT signaling was shown to be involved in the growth and maintenance of the prostate tumor stem cells in TRAMP mice [32]. These findings strongly suggest that PI3K signaling pathway plays a major role in the development of prostate cancer stem cells. The abundance of PI3K mediators in tumor stem cells would allow intake of increased glucose, providing the energy basis for their unseasing growth [20–22].

Histone acetylation regulates the interaction between nucleosomes and DNA, allowing the access of transcriptional factors to DNA [33–35]. The acetylation level is balanced by the acetyltransferases and deacetylases. The current study has documented the change of acetylation level at H3K9 in the spheres, corroborating our earlier observation that the spheres had altered histone acetylation as a result of PEITC treatment [7]. Further epigenomic studies of the PSC spheres may shed more light on the changes in the epigenetic landscape that may govern the androgen-mediated shuttling between sphere stem cells and the neuroendocrine features.

When monolayer cell culture transforms to 3D-culture like spheres, most of the proliferating cells are located in the outer cell layers, whereas the quiescent cells are located centrally [36, 37]. The quiescent cells in DU-145 prostate tumor spheroids was shown to have higher co- expression of p27 and p-glycoprotein [38]. Down-regulation of p27 in another study together with decreased p-glycoprotein sensitized ovarian cancer cells to taxol treatment, suggesting that p27 was involved in drug resistance [39]. These studies were consistent with our observation of p27 up-regulation in the LNCaP tumor spheres in this study. In a knock-in mouse model that prevented p27 from interacting with cyclins and CDKs, spontaneous tumors developed in multiple organs, along with an expansion of the stem cell population [40, 41]. Thus p27 has been proposed to have a duel role in tumorigenesis, one as a tumor suppressor in cyclin-CDK regulation of proliferation and two as an oncogene through a cyclin-CDK independent function. The p27 up-regulation in the sphere cells seen in this study could be one of the mechanisms for the development of the stemness and plasticity of the prostate cancer stem cells. Further experiments are clearly needed to confirm the role of p27 during the LNCaP prostate cancer sphere formation.

## Conclusions

Under PEITC treatment, there was up-regulation of p27. PI3K pathway members were activated whereas H3K9 acetylation was decreased in parallel. These findings correlated with the generation of prostate cancer stem cells (spheres) and suggest a distinct epigenome and signaling pathway from the LNCaP cells.

## Data Availability

The data and materials are available

## Abbreviations

PSC: Prostate cancer stem cells
PI3K: Phosphoinositol-3-kinase
AKT: Protein kinase B
p-AKT: Phosphorylated AKT or phosphor-AKT
HDAC: Histone deacetylases
PEITC: Phenethyl isothiocyanate
